# *p21* rs3176352 G>C and *p73* rs1801173 C>T Polymorphisms Are Associated with an Increased Risk of Esophageal Cancer in a Chinese Population

**DOI:** 10.1371/journal.pone.0096958

**Published:** 2014-05-12

**Authors:** Liang Zheng, Weifeng Tang, Yijun Shi, Suocheng Chen, Xu Wang, Liming Wang, Aizhong Shao, Guowen Ding, Chao Liu, Ruiping Liu, Jun Yin, Haiyong Gu

**Affiliations:** 1 Department of Cardiothoracic Surgery, The First People's Hospital of Changzhou and The Third Affiliated Hospital of Suzhou University, Changzhou, Jiangsu Province, China; 2 Department of Cardiothoracic Surgery, Affiliated People's Hospital of Jiangsu University, Zhenjiang, Jiangsu Province, China; 3 Cancer institute, Department of chemotherapy, People's Hospital Affiliated to Jiangsu University, Zhenjiang, Jiangsu Province, China; 4 Department of Orthopedics, Affiliated Hospital of Nanjing Medical University, Changzhou Second People's Hospital, Changzhou, Jiangsu Province, China; University of Texas MD Anderson Cancer Center, United States of America

## Abstract

**Objective:**

Esophageal cancer was the fifth most commonly diagnosed cancer and the fourth leading cause of cancer-related death in China in 2009. Genetic factors might play an important role in esophageal squamous cell carcinoma (ESCC) carcinogenesis.

**Designs and Methods:**

To evaluate the effect *p21*, *p53*, *TP53BP1* and *p73* single nucleotide polymorphisms (SNPs) on the risk of ESCC, we conducted a hospital based case–control study. A total of 629 ESCC cases and 686 controls were recruited. Their genotypes were determined using ligation detection reaction (LDR) method.

**Results:**

When the *p21* rs3176352 GG homozygote genotype was used as the reference group, the CC genotype was associated with a significantly increased risk of ESCC. When the *p73* rs1801173 CC homozygote genotype was used as the reference group, the CT genotype was associated with a significantly increased risk of ESCC. After Bonferroni correction, for *p21* rs3176352 G>C, the *p*_correct_ was still significant. For the other six SNPs, in all comparison models, no association between the polymorphisms and ESCC risk was observed.

**Conclusions:**

*p21* rs3176352 G>C and *p73* rs1801173 C>T SNPs are associated with increased risk of ESCC. To confirm the current findings, additional, larger studies and tissue-specific biological characterization are required.

## Introduction

As the fourth leading cause of cancer deaths and the fifth most common diagnosed cancer in China in 2009 [1], the 5-year-survival rate of esophageal cancer is very poor and accounts only 12.3% in 23 European countries [2]. More than 90% esophageal cancers are esophageal squamous cell carcinoma (ESCC). In addition to environmental risk factors such as smoking and heavy drinking, single nucleotide polymorphisms (SNPs) as genetic factors might play an important role in ESCC carcinogenesis [3].

Tumor suppressor protein p53 is frequently mutated in diverse types of cancers and is implicated in cell proliferation and tumor progression [4]. The *p53* gene is on chromosome 17p13.1. A well-studied *p53* polymorphism, Arg72Pro (rs1042522 C/G; R/P) has been reported to have functional significance [5], [6]. Compared with Arg wild-type protein, the Pro variant allele encoded protein is more efficient in inducing nuclear DNA repair genes expression [7]. Polymorphism *p53* rs1042522 G>C has been associated with risk of numerous kinds of cancers [8].

P21 (Waf1/Cip1/CDKN1A), a non-specific cdk inhibitor and a key mediator of G0-G1 cell cycle arrest, is upregulated by wild-type p53. p21 functions during gene repair and angiogenesis [9]. Cell cycle arrest at the G1-S phase restriction point is mediated through p21 up-regulation induced by p53, and the associated G1 cyclins-cdk2 complexes inhibition [10]. In p53-deficient cells, p21 interacts with proliferating cell nuclear antigen (PCNA) and causes both G1 and G2 cell cycle arrest [11]. By inhibiting PCNA-dependent DNA replication, p21 expression can suppress tumor growth and mismatch repair in vitro [12]. *p21* encodes a 21-kDa protein, is located on chromosome 6p21.2 and consists of three exons and two introns [13].

Tumor protein 53-binding protein 1 (TP53BP1) interacts specifically with *p53* and participates in both DNA repair and cell cycle control. By cooperating with damage sensors and signal transducers, TP53BP1 helps mediate the DNA damage checkpoint [14].

*P73* shares structural and functional similarities to *p53*. *p73* is located at 1p36.33, mapping to a region that is often deleted in cancers [15]. p73 activates transcription of p21- and p53-responsive genes, which participate in cell cycle control, DNA repair, apoptosis and inhibits cell growth in a p53-like manner by inducing apoptosis or G1 cell cycle arrest [16], [17]. This suggests that p73 has tumor-suppressor functions. Otherwise, the *p73* gene has some significant differences from p53. In contrast to p53-deficient mice, those lacking *p73* show no increased susceptibility to spontaneous tumorigenesis [18].

Genetic variations in the p53 pathway genes, such as *p21*, *p53*, *TP53BP1* and *p73*, may contribute to the development of ESCC. In a hospital-based case-control study, we performed genotyping analyses of eight functional *p21*, *p53*, *TP53BP1* and *p73* SNPs in 629 ESCC cases and 686 controls in a Chinese population.

## Materials and Methods

### Ethical approval of the study protocol

The data has been deposited in Supporting Information files. Regarding ethical conduct of research involving human subjects and/or animals, we complied with the World Medical Association Declaration of Helsinki. The review Board of Jiangsu University (Zhenjiang, China) approved this hospital-based case-control study. Written informed consent was provided by all subjects in the study.

### Patients and controls

Between October 2008 and December 2010, from the Affiliated People's Hospital of Jiangsu University and Affiliated Hospital of Jiangsu University (Zhenjiang, China), 629 subjects with esophageal cancer were recruited consecutively. By pathological means, all cases of esophageal cancer were diagnosed as ESCC. Exclusion criteria were: patients who previously had cancer; any metastasized cancer and radiotherapy or chemotherapy. 686 patients without cancer were matched to the cases with regard to age (±5 years) and sex, as controls. The controls were recruited from the above-mentioned two hospitals at the same time period. Most of the controls were being treated for trauma (including 612 trauma patients, 45 infectious disease patients and 29 hypertension patients).

Using a pre-tested questionnaire, trained interviewers questioned each subject personally and obtained demographic data information (e.g., age, sex) and related risk factors (such as tobacco smoking and alcohol consumption). Venous blood samples (2-mL) were collected after the interview from each subject. The definition of “smokers” was smoking one cigarette per day for >1 year. The definition of “alcohol drinkers” was consumption ≥3 alcoholic drinks a week for >6 months.

### Isolation of DNA, SNPs selection and genotyping by ligation detection reaction (LDR)

From whole blood, genomic DNA was isolated [19]. The 8 SNPs selection was based on previous published articles with functional consideration [20], [21], [22], [23]. With technical support from the Shanghai Biowing Applied Biotechnology Company, the samples were genotyped using the LDR method [24]. In 160 (12.17%) randomly selected samples with high DNA quality, repeated analyses were done for quality control.

### Statistical analyses

Using the *χ* ^2^ test, between the cases and controls, the distributions of demographic characteristics, selected variables, and genotypes of the *p21*, *p53*, *TP53BP1* and *p73* variants differences were evaluated. Using logistic regression analyses, the associations between the eight SNPs and risk of ESCC were estimated for crude ORs and adjusted ORs when adjusting for age, sex, smoking and drinking status. Because of the number of comparisons, the Bonferroni correction procedure was applied. By a goodness-of-fit *χ* ^2^ test, the Hardy-Weinberg equilibrium (HWE) was tested to compare the observed genotype frequencies to the expected ones among the control subjects. With SAS 9.1.3 (SAS Institute, Cary, NC, USA), all statistical analyses were performed.

## Results

### Characteristics of the study population

Cases and controls' characteristics are summarized in Table 1. By the *χ* ^2^ tests, the cases and controls are adequately matched on age and sex. Between the cases and the controls, significant difference was detected on smoking and drinking status, which is shown in Table 1. The primary information for eight genotyped SNPs was in Table 2. The concordance rates of repeated analyses were 100% except *p21* rs3176352 G>C (158/160, 98.75%). For all SNPs, minor allele frequency (MAF) in our controls was similar to MAF for Chinese in database. In the controls, for these eight polymorphisms, the observed genotype frequencies were all in HWE (Table 2).

**Table 1:** Distribution of selected demographic variables and risk factors in ESCC cases and controls.

Variable	Cases (n = 629)		Controls (n = 686)		*p* a
	n %		n %		
**Age (years)** mean ± SD	62.85 (±8.13)		62.58 (±7.89)		0.541
**Age (years)**					0.155
<63	310	49.28	365	53.21	
≥63	319	50.72	321	46.79	
**Sex**					0.185
Male	444	70.59	461	67.20	
Female	185	29.41	225	32.80	
**Tobacco use**					**<0.001**
Never	355	56.44	499	72.74	
Ever	274	43.56	187	27.26	
**Alcohol use**					**<0.001**
Never	428	68.04	526	76.68	
Ever	201	31.96	160	23.32	

a Two-sided *χ* ^2^ test and student t test; Bold values are statistically significant (*p* <0.05).

**Table 2:** Primary information for *p21*, *p53*, *TP53BP1* and *p73* polymorphisms.

Genotyped SNPs	*p21* rs2395655 G>A	*p21* rs1059234 C>T	*p21* rs3176352 G>C	*p21* rs1801270 C>A	*p21* rs762623 C>A	*p53* rs1042522 G>C	*TP53BP1* rs560191 G>C	*p73* rs1801173C>T
Chromosome	6	6	6	6	6	17	15	1
Gene Official Symbol	CDKN1A	CDKN1A	CDKN1A	CDKN1A	CDKN1A	TP53	TP53BP1	TP73
Function	5-UTR	3-UTR	intron region	missense	intron region	missense	missense	5-UTR
Chr Pos (Genome Build 36.3)	36753674	36761575	36760317	36759949	36753444	7520197	41555066	3588770
Regulome DB Score^a^	1f	4	4	3a	1b	5	1f	5
TFBS^b^	Y	—	—	—	Y	—	—	—
Splicing (ESE or ESS)	—	Y	—	—	—	—	Y	Y
miRNA (miRanda)	—	Y	—	—	—	—	—	—
miRNA (Sanger)	—	Y	—	—	—	—	—	—
MAF^c^ for Chinese in database	0.465	0.453	0.422	0.465	0.111	0.489	0.444	0.267
MAF in our controls (n = 686)	0.473	0.478	0.410	0.479	0.112	0.436	0.447	0.230
*p* value for HWE^d^ test in our controls	0.606	0.234	0.443	0.639	0.852	0.372	**0.009**	0.739
Genotyping method^e^	LDR	LDR	LDR	LDR	LDR	LDR	LDR	LDR
% Genotyping value	95.13%	95.13%	96.81%	98.63%	98.63%	96.35%	96.43%	96.81%

a http://www.regulomedb.org/;

b TFBS: Transcription Factor Binding Site (http://snpinfo.niehs.nih.gov/snpinfo/snpfunc.htm);

c MAF: minor allele frequency, *p73* rs1801173 C>T MAF is in CHB+JPT population;

d HWE: Hardy–Weinberg equilibrium;

e LDR: ligation detection reaction; Bold values are statistically significant (*p*<0.05).

### Associations between p21, p53, TP53BP1 and p73 polymorphisms and risk of ESCC and genotype combination analysis

When the *p21* rs3176352 GG homozygote genotype was used as the reference group, the GC genotype was not associated with the risk for ESCC; the CC genotype was associated with a significantly increased risk for ESCC (CC vs. GG: adjusted OR = 1.61, 95% CI = 1.18–2.20, *p* = 0.0030). In the dominant model, the *p21* rs3176352 GC/CC variants were not associated with the risk of ESCC, compared with the *p21* rs3176352 GG genotype. In the recessive model, when the *p21* rs3176352 GG/GC genotypes were used as the reference group, the CC homozygote genotype was associated with a 63% increased risk of ESCC (CC vs. GG/GC: adjusted OR = 1.63, 95% CI = 1.23–2.15, *p* = 0.0006) (Table 3).

**Table 3:** Logistic regression analyses of associations between *p21*, *p53*, *TP53BP1* and *p73* polymorphisms and risk of ESCC.

Genotype	Cases (n = 629)		Controls (n = 686)		Crude OR (95%CI)	*p*	Adjusted OR a (95%CI)	*p*
	n %		n %					
*p21* rs2395655 G>A								
GG	148	24.7	184	28.3	1.00		1.00	
GA	327	54.5	318	48.8	1.28 (0.98–1.67)	0.070	1.23 (0.94–1.62)	0.128
AA	125	20.8	149	22.9	1.04 (0.76–1.44)	0.798	1.00 (0.72–1.39)	0.982
GA+AA	452	75.3	467	71.7	1.20 (0.94–1.55)	0.150	1.16 (0.90–1.50)	0.256
GG+GA	475	79.2	502	77.1	1.00		1.00	
AA	125	20.8	149	22.9	0.89 (0.68–1.16)	0.381	0.87 (0.66–1.15)	0.332
*p21* rs1059234 C>T								
CC	172	28.7	170	26.1	1.00		1.00	
CT	321	53.5	340	52.2	0.93 (0.72–1.21)	0.604	0.88 (0.67–1.15)	0.334
TT	107	17.8	141	21.7	0.75 (0.54–1.04)	0.086	0.72 (0.51–1.00)	0.050
CT+TT	428	71.3	481	73.9	0.88 (0.69–1.13)	0.311	0.83 (0.64–1.07)	0.149
CC+CT	493	82.2	510	78.3	1.00		1.00	
TT	107	17.8	141	21.7	0.79 (0.59–1.04)	0.090	0.78 (0.59–1.04)	0.089
*p21* rs3176352 G>C								
GG	191	31.8	239	35.5	1.00		1.00	
GC	258	43.0	316	47.0	1.02 (0.79–1.31)	0.868	0.98 (0.76–1.27)	0.866
CC	151	25.2	118	17.5	**1.60 (1.18**–**2.18)**	**0.0026**	**1.61 (1.18**–**2.20)**	**0.0030**
GC+CC	409	68.2	434	64.5	1.18 (0.93–1.49)	0.166	1.15 (0.91–1.46)	0.255
GG+GC	449	74.8	555	82.5	1.00		1.00	
CC	151	25.2	118	17.5	**1.58 (1.21**–**2.07)**	**0.0009**	**1.63 (1.23**–**2.15)**	**0.0006**
*p21* rs1801270 C>A								
CC	179	29.1	182	26.7	1.00		1.00	
CA	322	52.3	346	50.8	0.95 (0.73–1.22)	0.672	0.89 (0.68–1.15)	0.373
AA	115	18.7	153	22.5	0.76 (0.56–1.05)	0.097	**0.72 (0.52**–**0.99)**	**0.044**
CA+AA	437	70.9	499	73.3	0.89 (0.70–1.14)	0.349	0.84 (0.65–1.07)	0.154
CC+CA	501	81.3	528	77.5	1.00		1.00	
AA	115	18.7	153	22.5	0.79 (0.60–1.04)	0.092	0.77 (0.59–1.02)	0.070
*p21* rs762623 G>A								
GG	480	77.9	537	78.9	1.00		1.00	
GA	129	20.9	136	20.0	1.06 (0.81–1.39)	0.667	1.06 (0.81–1.40)	0.675
AA	7	1.1	8	1.2	0.98 (0.35–2.72)	0.967	0.95 (0.33–2.70)	0.923
GA+AA	136	22.1	144	21.1	1.06 (0.81–1.38)	0.683	1.06 (0.81–1.38)	0.700
GG+GA	609	98.9	673	98.8	1.00		1.00	
AA	7	1.1	8	1.2	0.97 (0.35–2.68)	0.949	0.94 (0.33–2.67)	0.905
*p53* rs1042522 G>C								
GG	177	28.8	213	32.6	1.00		1.00	
GC	321	52.3	310	47.5	1.25 (0.97–1.61)	0.089	1.19 (0.92–1.54)	0.186
CC	116	18.9	130	19.9	1.07 (0.78–1.48)	0.663	1.04 (0.75–1.44)	0.810
GC+CC	437	71.2	440	67.4	1.20 (0.94–1.52)	0.144	1.15 (0.90–1.46)	0.273
GG+GC	498	81.1	523	80.1	1.00		1.00	
CC	116	18.9	130	19.9	0.94 (0.71–1.24)	0.648	0.93 (0.70–1.24)	0.639
*TP53BP1* rs560191 G>C								
GG	213	34.6	216	33.1	1.00		1.00	
GC	291	47.3	290	44.4	1.02 (0.79–1.31)	0.891	1.01 (0.78–1.30)	0.959
CC	111	18.0	147	22.5	0.77 (0.56–1.05)	0.092	0.76 (0.56–1.05)	0.093
GC+CC	402	65.4	437	66.9	0.93 (0.74–1.18)	0.558	0.92 (0.73–1.17)	0.515
GG+GC	504	82.0	506	77.5	1.00		1.00	
CC	111	18.0	147	22.5	**0.76 (0.58**–**1.00)**	**0.049**	0.76 (0.57–1.01)	0.055
*p73* rs1801173 C>T								
CC	311	51.8	401	59.6	1.00		1.00	
CT	251	41.8	235	34.9	**1.38 (1.09**–**1.74)**	**0.007**	**1.39 (1.10**–**1.76)**	**0.006**
TT	38	6.3	37	5.5	1.32 (0.82–2.13)	0.248	1.27 (0.78–2.07)	0.337
CT+TT	289	48.2	272	40.4	**1.37 (1.10**–**1.71)**	**0.006**	**1.37 (1.10**–**1.72)**	**0.006**
CC+CT	562	93.7	636	94.5	1.00		1.00	
TT	38	6.3	37	5.5	1.16 (0.73–1.85)	0.527	1.11 (0.69–1.79)	0.667
*p21* rs3176352 G>C and *p73* rs1801173 C>T combinations								
No risk variant genotype^b^	226	37.7	323	48.0	1.00		1.00	
Either one risk variant genotype	308	51.3	310	46.1	**1.42 (1.13**–**1.79)**	**0.0030**	**1.42 (1.12**–**1.80)**	**0.0035**
Both risk variant genotypes	66	11.0	40	5.9	**2.36 (1.54**–**3.62)**	**<0.0001**	**2.47 (1.60**–**3.82)**	**<0.0001**

a Adjusted for age, sex, smoking status and alcohol consumption;

b Risk variant genotype means *p21* rs3176352 CC or *p73* rs1801173 CT/TT; *p21* rs3176352 C vs. G OR = 1.26, 95% CI = 1.08–1.47, *p* = 0.0041; *p73* rs1801173 T vs. C OR = 1.26, 95% CI = 1.05–1.50, *p* = 0.0126. Bonferroni correction (number of mutiple test = 32) was performed to correct the *p* value (*p*_correct_); for *p21* rs3176352 G>C, the *p*_correct_ = 0.096 for CC vs. GG after adjusted for age et al., *p*_correct_ = 0.0192 for CC vs. GG/GC. For *p73* rs1801173 C>T, the *p*_correct_ = 0.202 for CT vs. CC after adjusted for age et al., *p*_correct_ = 0.195 for CT/TT vs. CC. For the rest 6 SNPs, *p*_correct_>0.05 in all comparison models; Bold values are statistically significant (*p*<0.05).

When the *p73* rs1801173 CC homozygote genotype was used as the reference group, the CT genotype was associated with a significantly increased risk for ESCC (CT vs. CC: adjusted OR = 1.39, 95% CI = 1.10–1.76, *p* = 0.006); the TT genotype was not associated with the risk of ESCC. In the dominant model, the *p73* rs1801173 CT/TT variants were associated with a significantly increased risk for ESCC (CT/TT vs. CC: adjusted OR = 1.37, 95% CI = 1.10–1.72, *p* = 0.006), compared with the *p73* rs1801173 CC genotype. In the recessive model, when the *p73* rs1801173 CC/CT genotypes were used as the reference group, the TT homozygote genotype was not associated with the risk of ESCC (Table 3).

When the *p21* rs1801270 CC homozygote genotype was used as the reference group, the AA genotype was associated with a significantly decreased risk for ESCC. When the *TP53BP1* rs560191 GG/GC genotypes were used as the reference group, the CC genotype was associated with a significantly decreased risk for ESCC (Table 3). Logistic regression analyses revealed that the *p21* rs2395655 G>A, *p21* rs1059234 C>T, *p21* rs762623 C>A and *p53* rs1042522 G>C polymorphisms were not associated with the risk of ESCC (Table 2). After the Bonferroni correction (number of mutiple test  = 32), for *p21* rs3176352 G>C, the adjusted *p* = 0.096 for CC vs. GG, adjusted *p* = 0.0192 for CC vs. GG/GC. For *p73* rs1801173 C>T, the *p*_correct_ = 0.202 for CT vs. CC after adjusted for age et al., *p*_correct_ = 0.195 for CT/TT vs. CC. For the rest 6 SNPs, in all comparison models, *p*>0.05.

When the *p21* rs3176352 CC genotype and *p73* rs1801173 CT/TT genotypes were considered as risk variant genotypes. When the no risk variant genotype carrier group was used as the reference group, the either one risk variant genotype carrier group (adjusted OR = 1.42, 95% CI = 1.12–1.80, *p* = 0.0035) and both risk variant genotypes carrier group (adjusted OR = 2.47, 95% CI = 1.60–3.82, *p*<0.0001) were associated with a significantly increased risk for ESCC.

### Stratification analyses on the p21 rs3176352 G>C and p73 rs1801173 C>T polymorphism and the risk of ESCC

To evaluate the effects of *p21* rs3176352 G>C genotypes on ESCC risk according to different age, sex, smoking and alcohol drinking status; we performed the stratification analyses. A significantly increased risk of ESCC associated with the *p21* rs3176352 G>C polymorphism was evident among all subgroups except in female patients after stratification (Table S1). A significantly decreased risk of ESCC associated with the *p73* rs1801173 C>T polymorphism was evident among older patients, female patients and patients who never drinking or smoking (Table S2).

## Discussion

In this hospital-based case-control study of ESCC, we found that the *p21* rs3176352 CC and *p73* rs1801173 CT/TT genotypes were associated with increased risk of ESCC; positive results were also observed in genotype combination analysis. To the best of our knowledge, this is the first positive association of *p21* rs3176352 G/C and *p73* rs1801173 C/T polymorphisms with ESCC risk.

P21 is a cyclin-dependent kinase inhibitor. It has been observed that in a wide variety of cancers, p21 expression is altered. At the G1 phase, the p21 protein disrupts cell cycle progression [25], [26]. Binding of the tumor suppressor protein p53 to the p21 promoter inducing p21 expression [27].

*P21* rs3176352 G/C (IVS2+16 G>C) is located in intron 2 of *p21*, 16 bp downstream from the splicing site. This C-to-G transition is predicted to affect the *p21* messenger RNA splicing [28]. Choi *et al.* demonstrated that *p21* rs3176352 G/C polymorphism appeared to be in linkage disequilibrium with Ser31Arg in a Korean population. Analysis of this haplotype for lung cancer susceptibility demonstrated a protective effect that was dependent on the number of variant alleles. In a previous study involving 80 esophageal cancer patients and 200 cancer-free controls from Ningxia Region of China, the *p21* rs3176352 G/C polymorphism was not associated with esophageal cancer risk [29]. A case-control study from northeastern Iran, with 126 cases and 100 controls, was carried out to detect associations of *p21* polymorphisms (rs1801270 and rs1059234) with ESCC risk [30]. The data suggested that these two *p21* polymorphisms, both alone and in combination, are not ESCC genetic susceptibility biomarkers, which agrees with our results.

P73, a p53 homolog, has some p53-like activities and plays an important role in modulating the cell cycle, apoptosis and DNA repair. In a high incidence region of China, *p73* polymorphisms were not associated with ESCC susceptibility [31]. However, our results are more reliable because of the higher numbers of cases and controls. *p73* rs1801173 C/T polymorphism merits further functional study to elucidate the etiology of this SNP and ESCC.

The frequencies of genetic polymorphisms often vary between ethnic groups. In the present Chinese study, the allele frequency of *p21* rs3176352 C was 0.410 in 686 control subjects, which is consistent with the values reported in the SNP database for the Chinese Han (0.422) and Japanese populations (0.455), higher than that of the Sub-Saharan African (0.233) population and African American population (0.250), and but lower than that of the European population (0.758). The allele frequency of *p73* rs1801173 T was 0.230 in 686 control subjects, which is consistent with the values reported in the SNP database for the CHB+JPT (Chinese Han+Japanese) populations (0.267), higher than that of the Sub-Saharan African (0.102) population and European population (0.150).

This case-control study had several limitations. First, because the patients and controls were enrolled from hospitals, inherent bias may have resulted in spurious findings. Second, the polymorphisms we studied may not provide a comprehensive view of *p21*, *p53*, *TP53BP1* and *p73* genetic variability. Fine-mapping studies are required. Third, because of the moderate sample size and absence of a validation cohort, the statistical power was limited. Finally, the viral infections and immune parameters information was not available, thus the power of our analyses was restricted.

In conclusion, our study provides strong evidence that *p21* rs3176352 G/C and *p73* rs1801173 C/T polymorphisms may contribute to ESCC risk. Tissue-specific biological characterization and replication studies with larger populations are required to confirm our findings.

## Supporting Information

Table S1 Stratified analyses between *p21* rs3176352 G>C polymorphism and ESCC risk by sex, age, smoking status and alcohol consumption.
(DOCX)

Table S2 Stratified analyses between *p73* rs1801173 C>T polymorphism and ESCC risk by sex, age, smoking status and alcohol consumption.
(DOC)

Data S1 Data of p21, p53, TP53BP1 and p73 polymorphisms.
(SAV)
