# Supplementary material for: p21 rs3176352 G>C and p73 rs1801173 C>T Polymorphisms Are Associated with an Increased Risk of Esophageal Cancer in a Chinese Population
Source: PLoS One. 2014 May 12;9(5):e96958. doi: 10.1371/journal.pone.0096958 (PMC4018405; doi:10.1371/journal.pone.0096958)
Supplement: Table S1 — Stratified analyses between p21 rs3176352 G>C polymorphism and ESCC risk by sex, age, smoking status and alcohol consumption. (DOCX) [file pone.0096958.s001.docx]

| Variable | *p21* rs3176352 G>C (case/control) ^a^ | | | |  | Adjusted OR ^b^ (95% CI); *p* | | | | |
| --- | --- | --- | --- | --- | --- | --- | --- | --- | --- | --- |
|  | GG | GC | CC | GC+CC |  | GG | GC | CC | GC+CC | CC vs. (GC+GG) |
| Sex |  |  |  |  |  |  |  |  |  |  |
| Male | 134/149 | 190/225 | 104/75 | 294/300 |  | 1.00 | 0.89 (0.65-1.22);  *p*: 0.482 | **1.55 (1.05-2.29);**  ***p*: 0.026** | 1.06 (0.79-1.41);  *p*: 0.712 | **1.66 (1.18-2.34);**  ***p*: 0.004** |
| Female | 57/90 | 68/91 | 47/43 | 115/134 |  | 1.00 | 1.18 (0.74-1.86);  *p*: 0.488 | 1.65 (0.97-2.82);  *p*: 0.066 | 1.33 (0.88-2.02);  *p*: 0.180 | 1.52 (0.94-2.45);  *p*: 0.086 |
| Age |  |  |  |  |  |  |  |  |  |  |
| <63 | 90/124 | 133/176 | 71/56 | 204/232 |  | 1.00 | 0.99 (0.69-1.44);  *p*: 0.968 | **1.68 (1.06-2.67);**  ***p*: 0.028** | 1.16 (0.82-1.64);  *p*: 0.405 | **1.69 (1.12-2.54);**  ***p*: 0.012** |
| ≥63 | 101/115 | 125/140 | 80/62 | 205/202 |  | 1.00 | 0.99 (0.69-1.43);  *p*: 0.974 | 1.50 (0.98-2.31);  *p*: 0.064 | 1.15 (0.82-1.61);  *p*: 0.414 | **1.51 (1.03-2.21);**  ***p*: 0.035** |
| Smoking status |  |  |  |  |  |  |  |  |  |  |
| Never | 115/180 | 132/221 | 86/91 | 218/312 |  | 1.00 | 0.93 (0.67-1.28);  *p*: 0.653 | 1.42 (0.97-2.08);  *p*: 0.073 | 1.08 (0.80-1.45);  *p*: 0.631 | **1.48 (1.05-2.08);**  ***p*: 0.026** |
| Ever | 76/59 | 126/95 | 65/27 | 191/122 |  | 1.00 | 1.02 (0.65-1.58);  *p*: 0.941 | **1.99 (1.12-3.54);**  ***p*: 0.019** | 1.23 (0.81-1.87);  *p*: 0.335 | **1.97 (1.19-3.27);**  ***p*: 0.008** |
| Alcohol consumption |  |  |  |  |  |  |  |  |  |  |
| Never | 138/190 | 170/234 | 96/95 | 266/329 |  | 1.00 | 0.97 (0.71-1.32);  *p*: 0.840 | 1.41 (0.98-2.04);  *p*: 0.068 | 1.10 (0.83-1.46);  *p*: 0.514 | **1.44 (1.03-2.00);**  ***p*: 0.032** |
| Ever | 53/49 | 88/82 | 55/23 | 143/105 |  | 1.00 | 0.94 (0.57-1.56);  *p*: 0.821 | **2.11 (1.12-3.97);**  ***p*: 0.021** | 1.20 (0.75-1.93);  *p*: 0.448 | **2.19 (1.26-3.79);**  ***p*: 0.006** |

^a^ The genotyping was successful in 600 (95.4%) ESCC cases, and 673 (98.1%) controls for *p21* rs3176352 G>C;

^b^ Adjusted for age, sex, smoking status and alcohol consumption (besides stratified factors accordingly) in a logistic regression model.
