# Supplementary material for: p21 rs3176352 G>C and p73 rs1801173 C>T Polymorphisms Are Associated with an Increased Risk of Esophageal Cancer in a Chinese Population
Source: PLoS One. 2014 May 12;9(5):e96958. doi: 10.1371/journal.pone.0096958 (PMC4018405; doi:10.1371/journal.pone.0096958)
Supplement: Table S2 — Stratified analyses between p73 rs1801173 C>T polymorphism and ESCC risk by sex, age, smoking status and alcohol consumption. (DOC) [file pone.0096958.s002.doc]

| Variable | *p73* rs1801173 C>T (case/control) ^a^ | | | |  | Adjusted OR ^b^ (95% CI); *p* | | | | |
| --- | --- | --- | --- | --- | --- | --- | --- | --- | --- | --- |
|  | CC | CT | TT | CT+TT |  | CC | CT | TT | CT+TT | TT vs. (CT+CC) |
| Sex |  |  |  |  |  |  |  |  |  |  |
| Male | 227/257 | 171/168 | 30/24 | 201/192 |  | 1.00 | 1.17 (0.88-1.55);  *p*: 0.287 | 1.38 (0.77-2.47);  *p*: 0.275 | 1.20 (0.91-1.57);  *p*: 0.202 | 1.30 (0.73-2.29);  *p*: 0.371 |
| Female | 84/144 | 80/67 | 8/13 | 88/80 |  | 1.00 | **1.99 (1.30-3.05);**  ***p*: 0.0015** | 0.98 (0.39-2.50);  *p*: 0.969 | **1.83 (1.21-2.75);**  ***p*: 0.0038** | 0.74 (0.30-1.86);  *p*: 0.527 |
| Age |  |  |  |  |  |  |  |  |  |  |
| <63 | 167/210 | 107/128 | 20/18 | 127/146 |  | 1.00 | 1.05 (0.75-1.48);  *p*: 0.769 | 1.36 (0.67-2.73);  *p*: 0.394 | 1.09 (0.79-1.51);  *p*: 0.605 | 1.33 (0.67-2.64);  *p*: 0.417 |
| ≥63 | 144/191 | 144/107 | 18/19 | 162/126 |  | 1.00 | **1.79 (1.28-2.50);**  ***p*: 0.0006** | 1.25 (0.63-2.47);  *p*: 0.532 | **1.71 (1.24-2.35);**  ***p*: 0.0011** | 0.97 (0.50-1.90);  *p*: 0.935 |
| Smoking status |  |  |  |  |  |  |  |  |  |  |
| Never | 169/294 | 145/173 | 19/25 | 164/198 |  | 1.00 | **1.45 (1.08-1.96);**  ***p*: 0.013** | 1.26 (0.66-2.40);  *p*: 0.478 | **1.43 (1.08-1.90);**  ***p*: 0.0142** | 1.08 (0.58-2.03);  *p*: 0.810 |
| Ever | 142/107 | 106/62 | 19/12 | 125/74 |  | 1.00 | 1.27 (0.84-1.91);  *p*: 0.255 | 1.17 (0.54-2.57);  *p*: 0.691 | 1.25 (0.85-1.85);  *p*: 0.256 | 1.07 (0.50-2.30);  *p*: 0.869 |
| Alcohol consumption |  |  |  |  |  |  |  |  |  |  |
| Never | 205/309 | 177/181 | 22/29 | 199/210 |  | 1.00 | **1.46 (1.10-1.94);**  ***p*: 0.008** | 1.05 (0.57-1.93);  *p*: 0.879 | **1.40 (1.07-1.84);**  ***p*: 0.0145** | 0.90 (0.49-1.63);  *p*: 0.718 |
| Ever | 106/92 | 74/54 | 16/8 | 90/62 |  | 1.00 | 1.19 (0.75-1.88);  *p*: 0.463 | 1.74 (0.70-4.35);  *p*: 0.236 | 1.26 (0.81-1.95);  *p*: 0.304 | 1.63 (0.66-3.99);  *p*: 0.288 |

^a^ The genotyping was successful in 600 (95.4%) ESCC cases, and 673 (98.1%) controls for *p73* rs1801173 C>T;

^b^ Adjusted for age, sex, smoking status and alcohol consumption (besides stratified factors accordingly) in a logistic regression model.
